# Designing the next‐generation therapeutic vaccines to cure chronic hepatitis B: focus on antigen presentation, vaccine properties and effect measures

**DOI:** 10.1002/cti2.1232

**Published:** 2021-01-15

**Authors:** Diahann TSL Jansen, Yingying Dou, Janet W de Wilde, Andrea M Woltman, Sonja I Buschow

**Affiliations:** ^1^ Department of Gastroenterology and Hepatology Erasmus MC University Medical Center Rotterdam Rotterdam The Netherlands; ^2^ Present address: Department of Viroscience Erasmus MC University Medical Center Rotterdam Rotterdam The Netherlands; ^3^ Present address: Institute of Medical Research Education Rotterdam Erasmus MC University Medical Center Rotterdam Rotterdam The Netherlands

**Keywords:** antigen presentation, chronic HBV infection, HBV, hepatitis B virus, HLA, therapeutic vaccination

## Abstract

In the mid‐90s, hepatitis B virus (HBV)‐directed immune responses were for the first time investigated in detail and revealed suboptimal T‐cell responses in chronic HBV patients. Based on these studies, therapeutic vaccination exploiting the antigen presentation capacity of dendritic cells to prime and/or boost HBV‐specific T‐cell responses was considered highly promising. Now, 25 years later, it has not yet delivered this promise. In this review, we summarise what has been clinically tested in terms of antigen targets and vaccine forms, how the immunological and therapeutic effects of these vaccines were assessed and what major clinical and immunological findings were reported. We combine the lessons learned from these trials with the most recent insights on HBV antigen presentation, T‐cell responses, vaccine composition, antiviral and immune‐modulatory drugs and disease biomarkers to derive novel opportunities for the next generation of therapeutic vaccines designed to cure chronic HBV either alone or in combination therapy.

## Graphical abstract

Therapeutic vaccination exploiting the antigen presentation capacity of dendritic cells to prime and/or boost HBV‐specific T‐cell responses was deemed highly promising but has not yet delivered its promise. In this review, we summarize what has been clinically tested in terms of antigen targets and vaccine forms, how the immunological and therapeutic effects were assessed and what clinical and immunological findings were reported. We combine this information with the most recent insights on HBV antigen presentation, T‐cell responses, vaccine composition, antiviral and immune‐modulatory drugs and disease biomarkers to derive novel opportunities for the next generation of therapeutic vaccines.

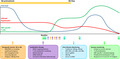

## Introduction

The hepatitis B virus (HBV) is a non‐cytopathic virus that specifically infects hepatocytes (reviewed by Lampertico *et al*.^1^ and Tu *et al*.^2^). The circular double‐stranded DNA composed viral genome is very small (3.2 kbp) and contains 4 overlapping open reading frames (ORF) encoding three forms of HBV surface antigen (HBsAg; small, medium and large), the viral capsid‐forming core (HBcAg) and its secreted form the e antigen (HBeAg), the viral DNA Polymerase (Pol) and lastly the non‐structural X protein that regulates viral protein transcription. These ORFs are translated directly from covalently closed circular DNA (cccDNA) residing as a mini‐chromosome in the nucleus of infected cells. Presumably to divert the immune system, infected hepatocytes not only secrete infectious viral particles, but also large amounts of HBeAg and HBsAg. The latter is secreted as empty, membrane enclosed, subviral particles. Soon after infection, the virus also integrates into the host genome, which contributes to hepatocellular carcinoma (HCC) development and viral persistence (reviewed by Tu *et al*.^2^). However, integration is not required for replication, which happens via cccDNA. Integration occurs from linearised viral DNA in which both Pol and HBcAg lose connection to their promotor, abrogating protein production. HBsAg and X, however, are likely readily produced from integrated DNA.

Although most adults can successfully clear an acute HBV infection, around 5% of adults and most perinatal infected infants develop a chronic HBV infection (cHBV). These patients are at risk of developing life threatening liver disease including HCC.^1^ Although direct oncogenic effects have been attributed to the X protein, cancer risk is also imposed by tissue damage resulting from the ongoing battle between HBV and the immune system. Initially, most patients experience a non‐inflammatory phase with high viral titres (HBeAg‐positive chronic infection (EPCI; Table 1), followed by an inflammatory phase (HBeAg‐positive chronic hepatitis (EPCH)) with immune‐mediated liver damage (i.e. ALT rise), fluctuating viral titres and ongoing expression of HBeAg. At some point, HBeAg expression may cease either or not in the presence of anti‐HBeAg antibodies and patients may enter a phase of partial immune control characterised by low viral titres (HBeAg‐negative chronic infection (ENCI)) in which most serum HBsAg derives from integrated DNA.^3^ Loss of HBeAg represents partial viral control, associates with HBV DNA decline and may precede HBsAg loss. Viral replication, however, may still return and lead to HBeAg‐negative chronic hepatitis (ENCH). A fifth but rare phase of HBV infection is characterised by absence of HBsAg in the serum with or without the presence of anti‐HBcAg and anti‐HBsAg immunoglobulins. Patients in this phase have normal ALT levels and detectable HBV DNA in serum and liver tissue.^1^ Currently, the only effective drugs are nucleos(t)ide analogs (NAs) that suppress viral replication, but offer no immune control and leave protein production intact and hence are recommended to be taken lifelong.^1^, ^4^ High costs limit NA availability and HCC risk is decreased, but not abrogated. Therefore, short‐term treatment that can establish full viral immune control, also called ‘a functional cure’ is highly demanded.^4^ A functional cure is reached when, in the absence of therapy, viral replication is maintained below detection limit and accompanied by the loss of serum HBsAg either or not in the presence of anti‐HBsAg immunoglobulins.

**Table 1:** Overview of previous and new nomenclature on the different phases of HBV infection^1^

Previous nomenclature	Immune Tolerance	Immune Active/Clearance	Inactive carrier	Immune Escape/Reactivation
HBV DNA	High	High	Low	High
HBsAg	High	High/intermediate	Low	Intermediate
HBeAg	Positive	Positive	Negative	Negative
Liver Function Tests (LFTs)	Normal	Abnormal	Normal	Abnormal
Liver disease	None/minimal	Moderate/severe	None	Moderate/severe
New nomenclature	HBeAg‐positive chronic infection (EPCI)	HBeAg‐positive chronic hepatitis (EPCH)	HBeAg‐negative chronic infection (ENCI)	HBeAg‐negative chronic hepatitis (ENCH)

Studies in chimpanzees have demonstrated that T cells represent a major factor associated with HBV immune control and clearance.^5^, ^6^ Concordantly, T‐cell responses are far more abundant and of higher quality in acute clearing patients as compared to cHBV.^7^, ^8^, ^9^ Hence, finding ways to activate, boost or introduce HBV‐directed T‐cell responses has been a focus of research for two decades. Ways to restore/obtain T‐cell responses include immune checkpoint blockade (ICB), therapeutic vaccination (TV) and adoptive T‐cell therapy.^10^ ICB will mostly act on existing responses while TV may also activate *de novo* responses. Here we will focus in detail on the clinical and immunological achievements of TV to harness T‐cell responses relying on antigen presentation by dendritic cells (DCs) and will explore remaining opportunities for TV. We will reflect on the implications of HBV antigen expression and HLA presentation as well as HBV disease stage. Furthermore, we will discuss recent developments with respect to immune and virus monitoring, vaccine composition and delivery and will touch upon combination therapies that could facilitate TV to cure cHBV.

## Priming and function of T cells in chronic HBV infection

Central to successful T‐cell priming and effector function is the process of antigen presentation by DCs and infected hepatocytes. To discuss the opportunities for TV we will first provide a brief outline of the ‘state of the art’ on HBV antigen processing and presentation by DCs, and the quality of HBV‐cognate T cells in cHBV.

### Dendritic cells

Dendritic cells recognise and take up pathogens or diseased, malignant or dying cells using a repertoire of pattern recognition receptors.^11^ Ingested material is processed by their intracellular machinery dedicated to antigen presentation on both HLA II (HLA‐DR/DP/DQ) and HLA I (HLA‐A/B/C) to prime (i.e. first time activate) CD4^+^ and CD8^+^ T cells respectively, supported by DC expressed co‐stimulatory receptors and cytokines. DCs excel in presentation of exogenous material on HLA I, which is called cross‐presentation. Because HBV does not infect DCs, the priming of HBV‐specific CD8^+^ T cells by DCs during HBV infection relies on DC cross‐presentation.^12^ Their unique T‐cell priming and stimulation capacity renders DCs of extreme importance for cHBV treatment as HBV‐clearing T‐cell responses could be initiated, boosted or qualitatively improved by ensuring that adequately matured DCs present the right HBV antigens.^13^ DCs can be used directly as a cellular vaccine, be targeted *in situ/vivo* by proteins, peptides, or particles designed to bind DC‐specific surface receptors or be targeted more passively by exploiting the unique cross‐presentation capacity of DCs.^14^, ^15^ The latter, for example, would be the case for vaccines based on whole proteins or synthetic long peptides (SLP).

Important for TV design is that DCs in cHBV need to be sufficiently operational, which is a highly debated topic. Many studies have described impairment of DCs to phenotypically mature or secrete cytokines directly after isolation from patient blood or livers, while others report DCs to be fully functional.^13^, ^16^, ^17^, ^18^, ^19^ Of note, many forms of TV are administered to the skin (intradermally or subcutaneously) or muscle and thus rely on intradermal and/or lymph node (LN) DC2 & DC1 for optimal CD4^+^ and CD8^+^ T‐cell priming respectively.^20^, ^21^ To our knowledge, functionality of intradermal or LN DC has not been studied in cHBV.

Thus far, both HBsAg and HBeAg have been demonstrated to suppress DCs *in vitro* (reviewed by Woltman *et al*.^22^). However, *in vivo* immune exhaustion, chronic inflammation, nutrient depletion, or cell stress is often seen in cHBV and could also affect DCs and confound results. Furthermore, inconsistencies between studies may have related to the source material (i.e. peripheral blood or liver), cHBV disease stage and/or treatment regime.

Despite the observations that T‐cell responses in general (i.e. also non‐HBV‐specific) may be dysfunctional in cHBV, there is currently no strong evidence that cHBV patients are impaired in their general ability to respond to pathogens or common vaccines, indicating that DCs are at least not greatly dysfunctional.^23^, ^24^, ^25^ Nonetheless, DCs may be of best quality in individuals with low viral load and liver inflammation (i.e. low ALT levels).^18^, ^22^, ^26^

### T cells

For TV design, it is pivotal to consider the quality of the T‐cell population. The state of HBV‐cognate T cells will be affected by the level and context of antigen presentation during priming of T cells, which is likely different for each viral antigen. Because Pol and X are expressed at a much lower level than HBsAg and HBcAg, that constitute the largest amount of protein in viral particles, HLA presentation from Pol and X is also likely to be lower.^27^, ^28^ However, few quantitative data on HBV protein expression are available. Furthermore, Pol and X are not (efficiently) secreted and therefore might reach DCs to a lesser extent. For these antigens hepatocytes are likely responsible for most of the T‐cell priming. Hepatocytes, however, lack proper co‐stimulatory signals to adequately prime CD8^+^ T cells and are also less able to activate CD4^+^ T cells.^29^ Hepatocyte primed dysfunctional CD8^+^ T cells, however, can be rescued by IL‐2.^30^ Because of these limitations, effective DC cross‐presentation of Pol and X peptides and subsequent priming of cognate T cells may be restricted to highest affinity peptides only. In contrast, average affinity peptides derived from HBsAg or HBcAg/HBeAg could already do the job.^31^ Conversely, secreted/virus‐contained HBeAg/HBcAg and HBsAg readily reach DCs and consequently may more abundantly prime T cells. HBsAg‐ and HBeAg/HBcAg‐cognate T cells for this very same reason could also be more prone to antigen overload‐driven deletion or exhaustion.^25^, ^32^, ^33^, ^34^ T‐cell deletion is most severe for HBsAg‐directed T cells and specifically for this antigen relates with age/exposure time rather than serum HBsAg levels.^35^ T cells recognising HBcAg are likely also affected by antigen overload, but partially restore after clearance of HBeAg, which is largely identical in amino acid sequence.^10^, ^23^

An effectively primed, high affinity T cell requires only few HLA‐peptide complexes to exert effector function.^36^ An outstanding question is how the hepatocyte HBV antigen expression levels that are needed to sustain the infection, relate to levels needed by CD8^+^ T cells to detect the infected cell. For endogenous HLA I presentation, protein abundance, intracellular location and turnover are important.^37^ Because HBsAg and HBcAg are crucial for viral particle formation, many more molecules of these proteins may be required for viral persistence than copies of Pol or X, which will also reflect on the presentation of these antigens on infected hepatocytes.^27^, ^38^ Yet, also HBcAg and HBsAg each have their own antigen presentation rules. Khakpoor *et al*.^39^ recently found that in ENCH patients presentation of a HBsAg epitope was spatially separated from a HBcAg epitope. It was postulated that high HBsAg presentation on cells from integrated DNA may serve as a decoy for T‐cell responses to spare *bona fide* infected hepatocytes (with possibly lower HBsAg presentation levels).

In line with differences in antigen presentation levels and cellular context during priming (e.g. by DCs or hepatocytes), three recent studies reported qualitatively different responses against HBcAg and Pol.^33^, ^34^, ^40^ HBcAg‐directed T cells were found less dysfunctional (i.e. with better *ex vivo* proliferation potential and cytokine secretion) and more of an effector memory (Tem) phenotype than those recognising Pol, which were mostly terminally differentiated effectors (Temra). This was, however, not associated with higher exhaustion marker expression on Pol‐cognate T cells. Exhaustion markers were found on all HBV‐recognising T cells regardless of protein specificity. Pol‐specific T cells rather expressed more KLRG1. Intriguingly, part of the Pol‐specific T cells displayed a naïve phenotype.^33^, ^34^, ^40^ Also in acute HBV infection PD‐1 expression was lower on Pol‐specific cells compared to HBcAg‐ and HBsAg‐specific T cells and Pol‐specific T cells were less functional *ex vivo*.^34^ Furthermore, also in acute patients HBcAg‐specific T cells were more of the Tem phenotype and Pol‐cognate T cells predominantly Temra. Presence of this phenotypic difference in both chronic and acute patients supports a mechanism independent of chronic disease which may include inherent differential antigen presentation and T‐cell priming (above; 34). Interestingly, for memory responses and specifically for generation of Tem over Temra CD4^+^ T cell help is required.^41^ In this regard evidence for an important role of CD4^+^ T cells in clearing cHBV is accumulating.^6^, ^42^, ^43^, ^44^ Besides helping CD8^+^ T cells, CD4^+^ T cells support B cell responses and might even harbour effector function themselves.^45^ CD4 effector action could occur indirectly via HLA class II presentation on nearby cytotoxic macrophages or even directly since hepatocytes under certain circumstances also express HLA class II.^46^, ^47^, ^48^ CD4 effector functions may thus potentially contribute to clear cHBV. Wrongly skewed CD4^+^ T cells, however, can also induce liver damage.^43^

## Past clinical studies on HBV therapeutic vaccination

In light of their ability to drive T‐cell responses against each of the viral proteins and of their clinical effect we have evaluated past clinical TV studies. TVs so far have been either based on HBV‐derived peptides or proteins (Table 2), genetically encoding (parts of) HBV antigens (Table 3) or based on *in vitro* loaded monocyte‐derived DCs (moDCs; Table 4). Many of these past trials mainly focused on viral persistence and did not very elaborately monitor immune responses, either only assessing antibody production and/or using T‐cell assays not discriminative for CD4^+^ or CD8^+^ T‐cell responses (e.g. IFNγ ELISpot). Initial TVs targeted only HBsAg. Over the years HBsAg remained a popular target. However, *ex vivo* (above) and *in vivo* (Tables 2, 3, 4) evidence suggests targeting HBsAg alone may not be very effective. Treatment of cHBV patients with peptide‐based GenVacB or Sci‐B‐Vac (Table 2), protein‐based YIC (Table 2) or DNA vaccines pCMV‐S2.S and ED‐DNA (Table 3) targeting HBsAg only, induced a decrease or even disappearance of HBV DNA, but always transiently.^49^, ^50^, ^51^, ^52^, ^53^, ^54^ HBsAg TV did induce HBsAg‐directed antibody responses in several cases, but ultimately without lasting effect on HBsAg levels.^50^, ^55^ Those HBsAg‐based TV trials that monitored T cells found that T‐cell responses to HBsAg were very low.^52^, ^56^ It is notable that the majority of peptide‐ and protein‐based vaccines targeting HBsAg used alum as adjuvant. While alum supports production of protective antibodies, it does not facilitate cytotoxic T‐cell responses.^57^ Other TVs, targeting HBsAg alone, however, were based on other vaccine platforms and/or were supported by different adjuvants, including AS02B, an oil in water, saponin and TLR4 ligand‐based adjuvant and DNA‐based TVs co‐encoding CpG (pCMV.S2.S) or IL‐12 and IFN‐ɣ (ED‐DNA).^52^, ^53^, ^54^ Each of these vaccines induced transient T‐cell responses except for ED‐DNA, which only induced T cells in patients displaying also a virological response to NA treatment started prior to ED‐DNA administration.^54^ Further of interest is that increasing the dosing frequency (of pCMV‐S2.S and YIC) did not necessarily augment immune responses or clinical effect, which for YIC was attributed to vaccine induced immune exhaustion.^52^, ^58^

**Table 2:** Peptide and protein‐based vaccines

Name	Composition	Groups, Route & follow‐up	Patients	Clinical effects	T‐cell response	Reference
Theradigm‐HBV	HBcAg18–27, Tetanus toxoid 830–843 + two × palmitic acid No adjuvant	Route: SC FU: 6 m	HBsAg^+^HBV DNA^+^HLA‐A2^+^ adults. *n* = 90	No Δ HBV DNA	Ag‐specific CD8^+^ T cells induced, but weaker than in healthy. TT response: Th1 in healthy and Th2 in cHBV. Poor CD4 = poor CD8 response	42, 59
GenHevac B	HBsAg, (pre‐S2) Adjuvant: Alum	GenHevac B or Recombivax vs no treatment Route: IM FU: 12 m	HBsAg^+^HBV DNA^+^ adults, 85% HBeAg^+^, therapy naïve. *n* = 118	No HBsAg loss. HBV DNA neg 6 m: 16% in vaccinated vs 3% in controls HBV DNA neg 12 m: 30% vs 24% in controls HBe loss/Anti‐HBe: 13% vs 4% in controls	Proliferative response: 5/17, all CD4^+^ T cells. Anti‐preS2 antibodies: 5/17 → 4/5 also preS2/HBsAg proliferative response	56, 121
		GenHeVAC B + IFNα‐2b vs IFNα‐2b Route: IM FU: 6 m	HBeAg^+^HBsAg^+^ HBV DNA^+^ children (2–13 y). *n* = 50	Transient Δ HBV DNA. Sustained anti‐HBeAg with HBV DNA neg; 13/25 (52%) in GenHevac B + IFNα vs 8/25 (32%) IFNα	No data	^49^
NASVAC (ABX203)	HBsAg, HBcAg Adjuvant: Alum	NASVAC vs no treatment Route: IN + SC FU: 48 w	Adults with high ALT, 61% HBeAg^+^. *n* = 28	7/11 HBeAg^−^ and 2/7 HBeAg^+^ became HBV DNA neg. Δ HBV DNA ↓ 2–5 log in 4/7 HBeAg^+^	*In vitro* HBsAg/HBcAgstimulated PBMCs and DCs from vaccinated patients produced more cytokines	^122^
		NASVAC vs PEG‐IFN Route IN + SC FU: 48–72 w	HBsAg^+^HBV DNA^+^ therapy naïve adults with ALT > ULN. *n* = 160	≈ Δ HBV DNA; anti‐HBeAg: NASVAC 5/14 (36%), Peg‐IFN 3/16 (19%)	No data	^123^
		NASVAC + NA vs NA > NA Stop Route: IN + SC FU: 24 w	HBeAg^−^HBV DNA^−^ adults with normal ALT > 2 y NA. *n* = 276	≈ viral relapse after NA stop	No data	124, 125
AS02B	HBsAg Adjuvant: AS02B	AS02B + LAM vs LAM Route: IM FU: 52 w	HBeAg^+^HBsAg^+^ adults with ALT 2–5× ULN. *n* = 161	≈ anti‐HBeAg, HBeAg loss, Δ HBV DNA: No HBsAg loss but anti‐HBs in 72% of AS02B + LAM vs 5% in LAM only	Tested: AS02B *n* = 6 and LAM *n* = 5. Proliferative response to HBsAg but not HBcAg at w7, but ↓ over time. IFNα and IL5 display same kinetics. No responses in Lam only	^55^
YIC	HBsAg complexed with HBIG Adjuvant: Alum	Route: IM YIC + ALUM vs ALUM FU: 24 w	HBeAg^+^ adults with high viral load and ALT 2–10× ULN. *n* = 20	Viral load ↓ + anti‐HBeAg: 5/10 YIC vs none in ALUM only. Anti‐HBs: 2/5 responders. Normal ALT: 3/5 responders	Cytokine production by HBsAg stimulated PBMCs in 4/5 responders at w24 and 1/5 at w44	^51^
		Route: IM YIC + ALUM (2 doses) vs ALUM FU: 24 w	HBeAg^+^HBsAg + anti‐HBe^−^ adults, HBV DNA > 10^5^ c mL^−1^, ALT 2–10× ULN. *n* = 237	More anti‐HBeAg in high dose YIC vs placebo (*P* = 0.03) (22% vs 9%), HBeAg loss 23 %, HBV DNA loss 37% at end of FU	No data	^126^
		Route: IM FU: 24 w	HBeAg^+^HBsAg^+^anti‐HBe^−^ adults, HBV DNA > 10^5^ c mL^−1^, ALT 2–10× ULN. *n* = 450	Anti‐HBeAg: YIC 14% vs alum 22%; ↓ viral load + ↓ HBsAg in all anti HBeAg responders; ALT norm in YIC only.	↓ serum IL‐17A in YIC group, anti‐HBeAg responders only	^58^
Sci‐B‐Vac	HBsAg (Pre‐S1, Pre‐S2, S) Adjuvant: Alum	Sci‐B‐Vac +/− LAM vs LAM Route: IM FU: 18 w	HBsAg^+^HBeAg^+^ adults, ALT 2–10× ULN. *n* = 180	≈ HBeAg loss/anti‐HBeAg (30% in both); anti‐HBs: 50/120 vaccine recipients only. ↓ HBV DNA in Sci‐B‐Vac +/− LAM at 12 m, not at 18 m	No data	^50^
DV601 (Theravax)	HBsAg, HBcAg Adjuvant: ISCOMATRIX	Dose escalation + ETV Route: IM FU: 99 d	HBeAg^+^ treatment naïve adults *n* = 8 HBeAg^−^ on NAs. *n* = 6	↓ HBV DNA + ↓ HBsAg + ↓ HBeAg in all dose groups. Anti‐HBsAg: 4/14, Anti‐HBeAg: 2/8 (both in high dose groups)	HBV‐specific proliferative responses in all. HBcAg‐specific IFNγ T‐cell response: 2/6 (both HBeAg^+^)	^95^
HepTcell (FP‐02.0)	Conserved regions of Pol, HBcAg, HBsAg (9 peptides) Adjuvant: IC31	Dose escalation +/− IC31 vs IC31 or placebo Route: IM FU: 6 w	HBeAg^−^ adults on ETV or TDF. *n* = 60	No effect on HBsAg	↑ HBV‐specific T‐cell responses vs baseline in vaccine + adjuvant groups. Responses mainly to pol	^84^
GS‐4774	Whole yeast cells expressing conserved regions of HBs, HBcAg and X	NA > GS‐4774 (dose escalation) + NA vs NA Route: SC FU: 48 w	HBsAg^+^ adults, HBV DNA < 2000 IU mL^−1^ on NA > 1 y. *n* = 178	No HBsAg loss, ≈ HBsAg ↓, HBeAg loss: 5/44 vs 0/7, anti‐HBeAg: 4/44 vs 0/7	*Ex vivo* ELISpot (w48): ↑ spot counts in GS‐4774 driven by HBcAg and x. ↑HBcAg response in HBeAg^−^ patients	^64^
		GS‐4774 (dose escalation) + TDF vs TDF Route: SC FU: 48 w	HBsAg^+^ adults, HBV DNA > 2000 IU mL^–1^, no antivirals > 3 m. *n* = 195	No HBsAg loss, No anti‐HBsAg, ≈ HBsAg ↓, HBeAg loss: 5/66 vaccinated vs 0/10 TFD only, HBeAg seroconversion: 3/66 vs 0/10	PMCS + 10 d OPP: ↑ IFNɣ, TNF and IL‐2 by CD8^+^ T cells, not CD4^+^ T cells by ICS. Response to pol, HBcAg and HBsAg, weak to x. *Ex vivo* ELISpot: response to HBcAg (50%) and pol (30%), not to HBsAg	^65^
		GS‐4774 + Nivolumab vs Nivolumab Route: SC FU: 24 w	HBsAg^+^, HBeAg^−^ adults on NAs > 1 y, HBV DNA < 20 IU mL^–1^ *n* = 24	HBsAg ↓ in 20/22 patients on 0.3 mg kg^−1^ nivolumab. Sustained HBsAg loss: 1 (on Nivolumab only). No added effect of GS‐4774	*Ex vivo* Fluospot (IFNγ + TNF): HBcAg and HBsAg‐specific T cells in 18/24 patients	^117^

SC, subcutaneous; IM, intramuscular; IN, intranasal; TT, tetanus toxoid; Ag, antigen; FU, follow‐up; vs, versus; >, followed by; ETV, entecavir; TDF, tenofovir; LAM, lamivudine; ALT, alanine aminotransferase; ULN, upper limit of normal; neg, negative; d, day; w, week; m, month; c mL^−1^, copies mL^−1^; NA, nucleot(s)ide analog; ↓, decrease; ↑, increase; Δ, change/difference; ≈ equal between groups.

**Table 3:** Genetic vaccines

Name	Vaccine composition	Groups, Route & follow‐up	Patient characteristics	Clinical effects	T‐cell response	Ref
pCMV‐S2.S	DNA vaccine encoding pre‐S and S Adjuvant: CpG in plasmid backbone	Route: IM FU: 15 m	cHBV adults with no response to IFNα or LAM, 6% HBeAg^−^ *n* = 10	↓ viral load: 2/10 ↓ of 50%: 4/10 HBeAg loss + anti‐HBeAg: 1	2/10 proliferative response to HBsAg; 1/10 response in *ex vivo* ELISpot; 5/10 after expansion with peptide pools for pre‐S2 and 10/10 for S. 2/10 increase in specific CD8^+^ T‐cell frequency in time. Mostly CD4 HBV response in non‐A2^+^. 2/10 anti‐preS2	52, 127
		pCMV‐S2S + NA vs NA > NA Stop Route: IM FU: 72 w	HBV DNA^−^ adults on NA treatment for 2 years. *n* = 70	Viral relapse: 97% (both groups w72) HBsAg ↓: no difference	*Ex vivo* ELISpot; no vaccine‐specific IFNγ production	^128^
HB‐100	Five plasmids encoding HBsAg, pre‐S1, pre‐S2, pol, HBcAg and X Adjuvant: co‐encoded IL‐12	LAM > HB‐100 + LAM Route: IM FU: 52 w	HBsAg^+^ adults with elevated ALT and high HBV DNA, 50% HBeAg^+^. *n* = 12	HBV DNA neg: 6/12 > HBeAg loss + anti‐HBeAg: 3/6 HBsAg loss + anti‐HBsAg: 1	*Ex vivo* ELISpot: 7/7 response to ≥1 HBV antigen. High spot number at end of treatment, but absent after FU. Cultured ELISpot; response up to 40 weeks after last vaccination. Response mainly CD4^+^ T cells	^62^
HB‐110	Improved HB‐100 encoding S and L HBsAg, HBcAg and Pol. Adjuvant: co‐encoded IL‐12	ADF > HB‐110 (dose escalation) + ADF vs ADF Route: IM FU: 48 w	HBsAg^+^HBeAg^+^ adults with elevated ALT and HBV DNA > 10^5^ *n* = 27	No HBsAg seroconversion. HBeAg seroconversion: 4/27 before HB‐110, 3 on HB‐110 stayed converted, 1/18 converted after HB‐110. ↓ viral load: trend	ELISpot; HBsAg, HBcAg and pol‐specific responses (high doses). ICS on 5 responders; multi‐functionality TNF, IFNγ, CD107 mainly in CD8^+^ T cells. Good T‐cell response at low DNA and ALT	^129^
ED‐DNA	Plasmid encoding M HBsAg Adjuvant: co‐encoded IL‐12 and IFNγ	LAM > LAM + ED‐DNA vs LAM Route: IM + electroporation FU: 72 w	HBsAg^+^HBeAg^+^ adults HBV DNA > 10^6^ c mL^−1^, ALT 2–10× ULN. *n* = 222	↓ HBV DNA: w48 & w64, not w72. More HBV DNA neg at w64, not w72. Low HBV DNA Subgroup analysis: ↑ HBeAg seroconversion in vaccine group.	PBMC + HBsAg for 9 days → IFNγ ELISA; 19/22 ED‐DNA + LAM 13/23 LAM only. IFNγ ICS CD8^+^ T cells after 6–8 h HBsAg 17/22 vs 10/23. *Ex vivo*/*in vitro* ELISpot comparable between groups.	^54^
DNA + MVA (TherVacB)	DNA plasmid encoding pre‐S2 and S. MVA encoding pre‐S2 and S No adjuvant	TherVacB regimen +/− LAM Route: IM + ID FU: 37 w	HBsAg^+^ males age 15–25, ALT < 88 IU mL^–1^ *n* = 72	No HBsAg loss No Δ HBV DNA HBeAg loss: 1	*Ex vivo* ELISpot; no vaccine‐specific IFNγ responses. ICS; no IFNγ production by CD4^+^ or CD8^+^ T cells. IFNγ was produced by CD16^+^ cells	^91^
TG1050	Non‐replicative adenovirus (Ad5) encoding HBcAg, pol and parts of HBsAg	TG1050 (dose escalation) vs placebo Route: SC FU: 52 w	HBsAg^+^ adults on ETV or TDF > 2 y UD HBV DNA, normal ALT, *n* = 48	Minor ↓ HBsAg No Δ HBcrAg	*In vitro* ELISpot after 10 day PP; 9/17 response to ≥ 1 antigen. 6/17 ≥ 2 antigens. Responses in highest doses. Response HBcAg > pol > HBsAg	^63^

>, followed by; ↑, increase; ↓, decrease; ADF, adefovir; Ag, antigen; ALT, alanine aminotransferase; ETV, entecavir; FU, follow‐up; ID, intradermal; IM, intramuscular; LAM, lamivudine; m, month; MVA, modified vaccinia Ankara; NA, nucleot(s)ide analog; neg, negative; pol, polymerase; pos, positive; SC, subcutaneous; TDF, tenofovir; UD, undetectable; ULN, upper limit of normal; vs, versus; w, week; Δ, change/difference.

**Table 4:** Dendritic cell vaccines

DCs type & handling	Groups, Route & follow‐up	Patient characteristics	Clinical effects	T‐cell response	Ref
Autologous moDCs pulsed with HBsAg	DC +/− LAM Route: SC FU: 1 y	HBsAg^+^HBeAg^+^ adults, HBV DNA > 10^5^ mL^−1^ *n* = 19	No HBsAg loss or anti‐HBsAg, ↓ HBV DNA: 11, HBeAg loss: 10/11, Anti‐HBeAg: 5/11, DCs + LAM; HBV DNA loss: 2/2, Anti‐HBeAg: 1/2	No data	^130^
Autologous moDCs pulsed with HBcAg18–27 and Pre‐S2 44–53 peptides	Route: IV FU: 48 w	HBsAg^+^ adults, no antiviral therapy > 6M *n* = 380	HBeAg^+^: HBeAg loss: 55/185 Anti‐HBeAg: 40/185 ↓ DNA > 2 log 10: 71/160 HBV DNA neg: 5/160 HBeAg^−^; HBsAg loss: 20/195, Anti‐HBsAg: 5/195, HBV DNA neg: 55/110	No data	^26^
Autologous moDCs pulsed with HBs183–191, HBcAg18–27 and pol575–583 and IL‐6, TNFα, IL1β & PGE2 matured	DCs +/− ETV vs ETV vs no therapy Route: IV FU: 2 y	HBsAg^+^HBeAg^+^ adults with HBV DNA > 10^4^ copies mL^−1^, no antiviral therapy > 1 y *n* = 80	No HBsAg loss or anti‐HBsAg, HBV DNA neg: 19/20 of DC + ETV vs 13/20 ETV only HBeAg loss: 11/20 DC + ETV vs 7/20 ETV only, Anti‐HBeAg: 8/20 DC + ETV vs 10/20 ETV only	No data	^61^
Autologous moDCs pulsed with commercial HBV vaccine containing HBsAg	Route: ID FU: 1 y	HBsAg^+^HBV DNA^+^ adults *n* = 5	Therapeutic potential not measured. No ↓ HBV DNA, Anti‐HBs: 2 of which 1 transient	No data	^131^

>, followed by; ↓, decrease; Ag, antigen; ETV, entecavir; FU, follow‐up; ID, intradermal; IV, intravenous; LAM, lamivudine; m, month; NA, nucleot(s)ide analog; neg, negative; SC, subcutaneous; vs, versus; w, week.

Considering the remaining T‐cell population in cHBV, targeting HBcAg, Pol or preferably even a collection of (partial) HBV antigens likely holds more promise than targeting HBsAg alone. Only one study targeted HBcAg alone using the immune dominant HLA‐A2 restricted HBcAg18–27 epitope attached to a Tetanus Toxid (TT) helper epitope (Theradigm‐HBV).^59^ Although without clinical effect, the Theradigm‐HBV study highlights that CD4^+^ T‐cell responses are important for the induction of primary, but not secondary CD8^+^ T‐cell responses and for CD8^+^ T‐cell longevity. Furthermore, TT‐directed CD4^+^ T cells and also PHA stimulated T cells from cHBV patients, but not HCV patients, were skewed towards IL‐15‐producing Th2 *ex vivo*. CD4^+^ T cells from healthy individuals, in contrast, produced the Th1 cytokines IL‐12 and IFNγ.^42^ These findings point to a more general Th skewing defect in cHBV patients. A mechanism could involve HBeAg present in the serum of Theradigm‐HBV treated patients that can drive unfavorable Th0/Th2 skewing.^60^

Besides single antigens, several different combinations of 2 to all HBV proteins have been targeted by TV either as whole proteins (i.e. alum supported NASVAC), DC *ex vivo* loaded with whole proteins or HLA‐peptides, yeast cells expressing whole protein (GS‐4774) or DNA (HB‐100, HB110; both co‐encoding IL‐12) or adenovirus encoding antigens (TG1050; Tables 2, 3, 4). Although also none of the multi‐antigen TV thus far yielded objective clinical effects, (transient) virological and/or T‐cell responses were induced by several. Peptide‐pulsed autologous DCs yielded a promising tendency towards increased HBeAg seroconversion especially when added to NA therapy. Unfortunately, no T‐cell responses were assessed.^61^ Responses against DNA vaccine HB‐100 (together with NA therapy) were mostly CD4^+^ and highest in patients with decreasing viral load. Induced CD4^+^ HBV‐specific central memory T cells persisted up to 40 weeks after the last injection.^62^ Of interest is that the HBeAg status related to the *in vivo* T‐cell response to HBcAg, in line with prior *in vitro* work.^23^ Also autologous DCs loaded with HBcAg and HBsAg performed better in HBeAg‐negative compared to positive patients.^26^ Adenovirus‐based TG1050 was tested primarily in HBeAg‐negative patients (only 3 out of 48 = 6.25% was HBeAg‐positive) and here strongest responses were detected against HBcAg by IFNγ ELISpot.^63^ Also for yeast‐based GS‐4774, IFNγ ELISpot for HBcAg was significantly more productive in the HBeAg‐negative patients (63.3% in HBeAg neg versus 35% in HBeAg pos).^64^ GS‐4774 (in combination with NAs) significantly induced T cells cognate for vaccine contained HBcAg (directly *ex vivo*) and HBsAg (after *in vitro* culture only) but not X. Despite effective CD8^+^ T‐cell induction, GS‐4774 started together with NAs in treatment naive patients did not yield any reduction in HBsAg over NAs alone. The lack of clinical effect may be explained by failure of GS‐4774 to induce CD4^+^ T‐cell responses. Important to note, however, is that GS‐4774 was based on HBV genotype D and most treated patients were Asian (likely infected with genotypes B and C) and 60% were HBeAg negative. T‐cell monitoring, however, was restricted to a small set of patients infected with genotype D which were all HBeAg negative. In the immune monitoring subgroup, both ethnicity and HBeAg status likely positively affected T‐cell response induction. Thus GS‐4774 may not to have been optimally matched to the patient population and the reported CD8^+^ T‐cell responses were not entirely representative for the cohort in which the clinical effect was assessed. Though, an important observation from the GS‐4774/Tenofovir study was that Pol‐specific cytokine‐producing T cells were triggered, while polymerase was not included in the vaccine.^65^ An explanation provided was a yeast adjuvant effect on Pol‐presenting DCs. In addition, it is possible that epitope spreading after cytolysis of infected hepatocytes, releasing Pol to DCs could have contributed.

Taken together, a wide variety of TV strategies have been developed and tested in clinical trials over the years with limited clinical success. Yet, the lessons learned from past clinical trials together with recent insight with respect to T‐cell responses in cHBV now set the stage for the design of new and improved TV strategies.

## Designing the next generation of vaccines

So far, despite induction of antibodies or T cells by some, no form of TV convincingly induced lasting viral control and HBsAg seroconversion in a significant number of patients. From other chronic infections and cancer, we can derive that immune suppressive mechanisms likely contribute highly to the lacking clinical effect and that combination with drugs lifting these mechanisms could be key also in cHBV.^66^, ^67^ However, such drugs will depend on the size and quality of the immune response at the time of administration. Therefore, to offer combination therapies the best starting point it is still paramount to carefully select the target antigen(s), vaccine platform, adjuvants, route of administration and patient population for TV. Furthermore, to allocate the best opportunities for combination (immune) therapy, both immunological and viral response monitoring alongside future TV clinical trials needs great detail and careful design.

### Vaccine target antigens

To limit competition between vaccine antigens their selection needs to be well‐considered. The off‐target Pol response seen upon GS‐4774 vaccination indicates that not all HBV proteins need to be targeted to get a broad polyclonal response. One or a few well‐chosen hits could kick‐start a self‐propagating response. Pinpointing exactly which T‐cell responses can trigger such a response to drive viral clearance has proven difficult. Recently, Bertoletti and co‐workers demonstrated that prior to stopping NA therapy, non‐flaring patients displayed higher numbers of HBcAg‐ and/or Pol‐specific T cells.^68^ Furthermore, Newell and colleagues identified HBcAg‐cognate T cells to associate with viral control, both between cHBV patient groups (i.e. with more, fitter and public TCR carrying T cells in resolver and ENCI over EPCH patients), and longitudinally towards HBeAg clearance and anti‐HBeAg seroconversion (i.e. a higher quality of HBcAg‐specific T cells at NA start^40^). Lastly, Deng *et al*.^43^ found that during a flare IFNɣ‐producing CD4^+^ T cells specific for HBcAg and HBsAg associated with HBeAg and HBsAg clearance respectively. Importantly, TNFα‐producing HBcAg‐specific CD4^+^ T cells rather associated with liver damage. While these studies indicate that boosting/triggering CD8^+^ T‐cell responses against HBcAg and Pol and CD4 responses against HBcAg and HBsAg may be most interesting, evidence is still circumstantial. To rationally design TV we believe it is still pivotal to gain mechanistic insight into why specific responses are preferred over others. Knowing for example which viral HLA I epitopes are presented even at low viral replication would greatly contribute. Furthermore, despite recent efforts into this direction, still more detailed data are required on (the parts of) viral antigens for which a salvageable CD8^+^ T‐cell population remains in most cHBV patients. For now, HBcAg and Pol may have the best cards. If, however, HLA I presentation of Pol by infected hepatocytes is truly very inefficient, one could argue that Pol can still support viral replication at ‘sub‐HLA’ expression levels. Yet, Pol‐directed immune responses could still suppress viral replication to a manageable viral load and give opportunity to responses of other specificities to clear the infection.^68^ Of note, HBsAg and X remain of special interest as targets in HBV‐derived HCC as these proteins are expressed from integrated DNA also in tumor cells.^69^

Regardless of the chosen target antigen(s), it is now clear that to obtain effective and long‐lasting CD8^+^ T‐cell responses, CD4^+^ T‐cell help is required and thus both CD4 and CD8 epitopes should be included in a vaccine.^21^ Because the same DC1 in the lymph node likely needs to present both CD4 and CD8 epitopes, and because antigen‐specific CD4^+^ T‐cell help is also important for recruitment to and proliferation of CD8^+^ T cells at the target tissue, physical linkage of these epitopes is desired.^21^, ^70^, ^71^ Finally, genetic variability between HBV genotypes demands either making genotype specific or personalised vaccines, or rather a focus on conserved parts of viral antigens.^72^

### Vaccination platform

To pinpoint which platforms have highest T‐cell activating potency, we can learn from other disease settings targeting viral‐ or neoantigens.^73^, ^74^, ^75^, ^76^, ^77^ In chronic human papilloma virus (cHPV)‐infected patients with pre‐malignant lesions, both SLPs and DNA vaccines could clear viral disease and lesions in a large proportion of patients.^73^, ^74^, ^75^ Importantly, the strength of the SLP‐induced responses directly correlated with clinical effect.^78^ Thus, both SLPs and DNA/RNA vaccination have proven efficacy to induce a virus clearing cellular response and for this reason are of special interest. DNA or RNA vaccines can support HLA I presentation by DCs via co‐translational peptide loading (rather than cross‐presentation) potentially benefiting CD8^+^ T‐cell responses (reviewed by Pardi *et al*.^79^). Both also readily induce CD4^+^ T‐cell responses.^62^, ^76^ Further considerations for vaccine platform choice are that whole proteins are ineffectively cross‐presented and thus not very potent to induce CD8^+^ T‐cell responses.^80^ Shorter protein fragments or SLPs are better cross‐presented and may harness responses also against (potentially less exhausted) subdominant CD8 epitopes.^81^ Using HLA‐restricted exact CD8^+^ T‐cell epitopes for TV is likely not preferred as it can lead to tolerance as a result of presentation by non‐professional APCs or T cells.^20^ CD4^+^ T‐cell presentation and processing of HLA II epitopes is more promiscuous and both whole proteins and shorter fragments could be effective.^21^ Furthermore, for protein‐based TV, particulation may facilitate CD8^+^ T‐cell induction and Th1 skewing.^82^ Currently, SLP‐based vaccines for cHBV are in (pre‐) clinical development by us and others.^13^, ^72^, ^83^, ^84^ In addition, several other protein‐based forms of antigen delivery are currently assessed in pre‐clinical studies: modified cell‐permeable HBV capsids are tested to deliver HBV antigens to the DCs cytoplasm to favor HLA I presentation.^85^, ^86^ Furthermore, a chimeric fusion protein consisting of the Fc fragment of a murine antibody and HBV antigens is tested targeting HBV antigens to DCs via Fcɣ and mannose receptors.^87^

Possibly, one platform alone may not do the job. As such heterologous prime‐boost vaccine regimens have proven to be an effective strategy to induce both humoral and cellular immune responses.^88^, ^89^, ^90^ A HBsAg‐containing DNA vaccine prime combined with a modified vaccinia virus Ankara (MVA) boost has already been tested in the clinic (TherVacB^91^). Improvements of this strategy by targeting HBcAg instead HBsAg and addition of a TLR‐ligand (i.e. CpG) are currently in the pre‐clinical phase as are other combinations of prime and boost formulations targeting multiple HBV antigens such as chimpanzee adenoviral and MVA vectors or attenuated vesiculostomatitis virus‐based platforms.^90^, ^92^, ^93^

### Adjuvants

Adjuvants are in many cases crucial to prevent presentation of antigens in a tolerogenic context. It is important, however, to tailor the adjuvant to the mechanism of action and desired outcome of the TV platform. As indicated, alum is not the best choice when a cellular response is aimed for. Protein‐ and peptide‐based TV may be best supported by an adjuvant triggering DC maturation during antigen uptake and thus linking the two can be favorable.^83^, ^94^ The cross‐presentation supporting adjuvant ISCOMATRIX is being tested as adjuvant for an HBsAg/HBcAg containing vaccine (DV601) and a preliminary report indicated induction of cellular responses.^95^ Alongside SLP vaccines in (pre‐) clinical development, TLR9 ligand IC31 (i.e. for HepTcell) and TLR2 and TLR3 ligands are used to improve vaccine effect.^83^, ^84^, ^96^ While TLR2 and TLR3 can be readily found on the myeloid DCs that are likely needed for TV effect, this is different for TLR9 that in humans is predominantly found on pDCs and B cells.^14^ TLR9 ligands, however, could still support cross‐presentation via the secretion of Type I interferons.

RNA and DNA vaccine platforms require a completely different adjuvant strategy as the target antigen first needs transcription and/or translation. Nucleic acid‐based platforms potentially contain intrinsic adjuvant capacity triggering nucleic acid recognising PRR. This may also hamper their effect because an IFN induced translational shutdown can impair vaccine encoded antigen expression. Most recent RNA‐based vaccines have been optimised to prevent this. Genetic vaccines benefit most from co‐encoded cytokines (such as IL‐12 for HB100 and HB110), co‐stimulatory agonists or constitutively signalling receptors.^79^

### Route of administration

Most cHBV vaccines thus far relied on intramuscular administration, some on subcutaneous injection and one on intranasal delivery (Tables 2, 3, 4). While intramuscular and subcutaneous routes are practical, well‐tolerated and equally adequate to trigger IgG1 antibody responses, they do not optimally trigger Th1 responses.^97^ More effective may be intranodal or intradermal vaccination that either directly bring the antigen to the site of action or use the vast network of skin resident migratory DCs for transport to the lymph nodes respectively.^98^, ^99^ Intradermal vaccination and even more so intranodal vaccination are effective at lower dose as compared to intramuscular or subcutaneous routes.^98^, ^100^ Intranodal delivery was recently used in a highly promising RNA vaccination strategy applied to melanoma patients.^76^ A drawback of these routes of administration is that they require more training.

### Patient population

cHBV disease stage likely greatly affects treatment outcome. Several studies show that in HBeAg‐negative patients, HBcAg‐cognate T‐cell activation is more effective and immune fitness greater as compared to HBeAg‐positive patients, which is associated with higher mutation driving immune pressure.^23^, ^62^, ^64^, ^101^ Mechanism could involve less exhaustion of HBcAg‐specific T cells due to the absence of HBeAg, and relief from unfavorable Th skewing and/or induction of myeloid suppressor cells by HBeAg.^60^, ^102^ Furthermore, T‐cell responses are better in patients with low viral load/ALT and DCs are more fit.^18^, ^22^, ^25^, ^26^, ^32^, ^103^ Low viral load may arise naturally or can be achieved by NA therapy. NA therapy, however, only transiently improves T cells’ responses, possibly reflecting initial rescue from antigen overstimulation and subsequent memory retraction of T cells.^104^

While at first patients with high viral load and ALT were mostly treated with TV, later co‐administration with NAs became more common. For safety reasons and because T cells may be most salvageable, nowadays many trials start TV treatment after a longer period of NA treatment and/or restrict patient inclusion to those with low/absent viral load. Important to consider, however, is that especially ENCI patients on NA therapy may have little viral activity remaining and serum HBsAg mostly derives from integrated DNA.^3^ Therefore, a beneficial effect of TV may not become immediately apparent and may require NA discontinuation to revive virus replication and antigen presentation. This to further boost the induced immune response and allow it to clear the virus.^105^ Although NA stop was not successful following HBsAg‐based vaccines (i.e. NASVAC and pCMV.S2.S), NA stop has not yet been attempted following vaccines targeting HBcAg or Pol and/or vaccines that were successful in triggering cellular immune responses (above). The latter is important because in particular HBcAg‐ and/or Pol‐directed T‐cell responses could limit flares upon NA discontinuation.^68^ Such T cells were deemed necessary to be present already prior to the flare, suggesting that TV prior to NA discontinuation could give HBcAg‐ and Pol‐specific T cells a head start on a possible revival of HBV replication. Although preventing a flare is surely a safer option on the short term, limited ‘flaring’ may accompany a beneficial HBV‐directed immune responses and ultimately HBsAg seroconversion giving a long‐term benefit.^4^ NA re‐treatment rules, however, should be carefully designed when considering stopping NAs.

Despite high DNA levels, young patients without liver damage could still represent an interesting target population as they are endowed with a more sizable and variable HBV‐directed T‐cell population and may thus respond better to TV.^35^, ^106^ Yet also (or even in particular) for this population suppression of viral replication with prior NA therapy will likely be needed to fully harness T cells and to warrant safety. A requirement for low viral load could complicate patient inclusion as these patients most often lack clinical symptoms and are not by default treated with NA therapy.^1^

### Outcome measures

To exploit TV for cHBV both viral and immunological responses need close and detailed monitoring. For past trials, monitoring was often restricted to measuring HBsAg levels and HBsAg‐directed antibodies (Tables 2, 3, 4). We now know these parameters do not optimally reflect vaccine efficacy as both the clearing of HBsAg and the development of HBsAg‐directed antibodies are relatively ‘late’ events on the road to viral clearance. Monitoring should perhaps focus more on obtaining a broad and qualitative view of vaccine immunogenicity and on finding more subtle signs of improved viral immune control. This is needed to reveal where a vaccine has an effect and to find out where further improvement of design and/or combination (immune) therapy could be applied to tip the balance.

From clinical successes in cHPV and cancer we know that the strength of the induced immune response is very important and that ultimately success may depend on counteracting T‐cell dysfunction.^66^, ^76^, ^77^, ^78^, ^107^ Therefore, a broad and antigen‐specific overview of induced T‐cell responses is needed and as much detail as possible on the size and quality (e.g. skewing, level of dysfunction) of these responses. Furthermore, it is important to take multiple baseline samples to correct for ‘normal’ fluctuations and to use pre‐ and well‐defined response measures.^78^, ^105^ Assays to follow the evoked immune response include traditional ELISpots both directly *ex vivo* and after short (i.e. 1–3 weeks) culture with vaccine constituents and/or overlapping peptide pools of preferably the separate HBV antigens, T‐cell proliferation assays and finally extensive immune phenotyping and/or single cell RNA sequencing of T cells cognate for (pools of) epitopes using advanced HLA‐multimer technologies.^33^, ^34^, ^40^, ^78^ Wherever possible not only the peripheral, but also the hepatic compartment should be sampled. Rational attempts should be made to correlate the size, quality and characteristics of the immune responses to effects of the vaccine on viral control. For the latter, besides traditional parameters (i.e. HBsAg, HBeAg and HBV DNA) several alternative markers have recently surfaced. These are important because HBsAg from integrated DNA may shield any effect on HBsAg from productively infected cells, while NA therapy (inhibiting Pol to synthesise relaxed circular (rc)DNA from pregenomic (pg)RNA) may mask effects on viral DNA.^3^, ^4^ One of the novel markers is serum pgRNA, which in NA treated patients may better represent cccDNA transcriptional activity than serum DNA.^4^, ^108^, ^109^, ^110^ A second novel marker is HBV core related antigen (HBcrAg), which represents a combination of proteins produced from the Core/Precore ORF and was found to correlate better with cccDNA transcriptional activity than HBsAg, HBV DNA or pgRNA.^111^, ^112^ Positive effects of TV on HBcrAg and pgRNA could thus represent an important sign of emerging viral immune control. Moreover, these markers could indicate whether TV can precondition patients for other forms of therapy or NA discontinuation. Lastly, although in need of validation, serum cccDNA could be of interest as it may reflect killing of cccDNA containing hepatocytes.^113^

### Other opportunities for combination (immune) therapy

T‐cell responses to HBV antigens boosted/induced by TV may be further improved by ICB, co‐stimulatory agonists and drugs altering T‐cell metabolism.^10^, ^24^, ^32^, ^114^, ^115^ As outlined above, recent studies strongly indicate that for each viral protein the mechanisms and deficits of T‐cell priming are different and likewise that different strategies may be required to activate, boost or rescue cognate T cells specific for each of these proteins. For example one could argue that for (part of the) HBcAg‐specific CD8^+^ T‐cell responses, ICB alone may already be effective in some patients, while for Pol‐specific CD8^+^ T cells support of T‐cell priming could first be needed.^33^, ^34^, ^116^, ^117^ Such support could be delivered either by TV or by an *in situ* vaccine effect of hepatocytes killed by T cells of a different specificity (e.g. HBcAg) as was seen in the GS‐4774 trial.^65^

Although still requiring a randomised large‐scale follow‐up, a recent study combining yeast‐based GS‐4774 together with ICB (anti‐PD1) showed HBsAg decline in several patients and one patient achieving functional cure. In this small (suboptimal, mostly Asian and Polynesian) cohort the induced HBsAg decline was not further facilitated by GS‐4774 pretreatment.^117^ Nonetheless, combination of (future generations of) TV with other ways of lifting immune suppression are worth exploring as is also highlighted by recent studies in HPV‐induced malignant disease and melanoma, where clinical responses to SLP/RNA vaccination were facilitated by depletion of myeloid derived suppressor cells or by combination with ICB.^77^, ^107^, ^118^

Also of high interest to combine with TV and/or drugs to lift immune suppression are therapeutic modalities specifically aimed at elimination of the vast load of HBsAg by interfering RNAs.^3^ In mice it has been demonstrated that knockdown of HBV antigens by siRNA in combination with TV (but not siRNA alone or TV alone) increased HBV‐specific effector T‐cell numbers resulting in loss of serum HBsAg, HBeAg and HBV DNA, development of high anti‐HBs titres and anti‐HBeAg seroconversion indicating a cure of HBV infection.^119^ So these data indicate that reducing HBsAg levels using siRNAs support the effectivity of the TV. As HBsAg levels do not seem to directly affect global or HBV‐specific T‐cell responses the support of siRNA may be explained by effects of HBsAg on sustaining HBV replication and/or on B cell responses, which likely also contribute to viral clearance.^35^, ^120^ A clinical study in humans and chimpanzees with siRNA targeting HBsAg was unfortunately not successful as it did not affect HBsAg expression from integrated DNA.^3^ Clinical results of follow‐up studies with siRNA that also silences this HBsAg are now eagerly awaited.

## Concluding remarks

**Figure 1:**
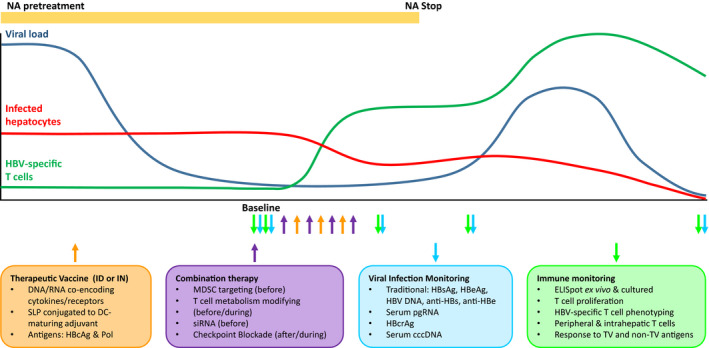
Considerations for the next generation of TV. Before TV administration, viral load may need to be reduced by NA and/or siRNA treatment. When viral load (and ALT) are stably low, TV can be given preferably intradermal (ID) or intranodal (IN). To further improve T‐cell effector function, TV can be combined with suppressive myeloid cell (MDSC) targeting drugs (given before TV), T‐cell metabolism modifying drugs (before or during TV) or checkpoint blockade (during or after TV). As a ‘natural’ booster, NAs can be stopped to increase viral antigen presentation boosting HBV‐specific T cells *in situ* to drive clearance of remaining infected hepatocytes. Adequate monitoring of the viral as well as immunological response is pivotal to evaluate vaccine efficacy. Multiple baseline samples are desired. In addition to traditional viral markers also, novel markers may be monitored. Immune monitoring should be as detailed as possible on peripheral blood samples, but whenever possible also on hepatic samples. Lines provide a schematic indication of the development of the indicated parameter over time. Coloured arrows match the boxes below the graphs and schematically indicate preferred moments of intervention or monitoring.

Taken together, based on results from other diseases we might deduce that SLP and nucleic acid‐based vaccines may have a high potency in the fight against cHBV. Their full potential, however, may only be exploited when the right (parts of) HBV antigens are targeted, when the latest mechanistic insight into *in vivo* vaccine handling by APCs at different anatomical sites are considered and when adjuvant and route of administration are rationally matched to the platform and the desired immunological outcome. For optimal effect of vaccination, it is likely essential to take a stepwise approach, first reducing viral load by NA therapy and/or by siRNA‐based therapeutic modalities (Figure 1). Combination or follow‐up with ICB, metabolism‐altering drugs or those targeting suppressive myeloid cells may be useful to harness both existing responses and to further boost TV‐induced responses. Ultimately, patients with effective T cells and temporarily alleviated HBsAg levels may benefit from a well‐timed, controlled and monitored NA stop to obtain a natural ‘booster’ for the immune system and allow immune clearance of the remaining infected cells.

## Conflict of interest

As stated above the authors collaborate and are co‐funded by ISA Pharmaceuticals B.V., Leiden, The Netherlands. The authors declare no further conflicts of interest.

## Author contributions

**Diahann TSL Jansen:** Conceptualization; Visualization; Writing‐original draft; Writing‐review & editing. **Yingying Dou:** Supervision; Visualization; Writing‐review & editing. **Janet de Wilde:** Conceptualization; Writing‐review & editing. **Andrea M. Woltman:** Funding acquisition; Supervision; Writing‐review & editing. **Sonja I Buschow:** Conceptualization; Funding acquisition; Supervision; Writing‐original draft; Writing‐review & editing.
